# Gradenigo's Syndrome and Labyrinthitis: Conservative versus Surgical Treatment

**DOI:** 10.1155/2018/6015385

**Published:** 2018-07-30

**Authors:** Ahmad Al-juboori, Amira Nasser Al Hail

**Affiliations:** ^1^Otorhinolaryngology, Head & Neck Surgery (ORL-HNS) Department, Al Wakra Hospital, Hamad Medical Corporation, Doha, Qatar; ^2^Weill Cornell Medical College, Ar-Rayyan, Qatar

## Abstract

**Background:**

Extracranial intratemporal complications of chronic suppurative otitis media (CSOM) are extremely rare. Gradenigo's syndrome is defined as a clinical triad of otitis media, severe pain originating from the trigeminal nerve, and ipsilateral sixth cranial nerve palsy.

**Case Report:**

A 61-year-old man presented with chronic left ear discharge, left-sided headache, diplopia associated with vertigo, tinnitus, and hearing impairment. MRI with contrast showed asymmetrical signal changes in the bilateral petrous bone with reduced enhancement on the left with high suspicion of petrositis; in the context of chronic tympanomastoiditis, there was a 10 × 4 mm enhancing lesion in the left internal auditory meatus involving the 7th-8th nerve complex. The patient was treated conservatively with local and systemic antimicrobial agents, he had satisfactory response and improvement regarding symptoms of ear discharge, vertigo, and diplopia, but there is no remarkable response regarding hearing loss and tinnitus.

**Conclusion:**

Although there is little evidence to support the use of conservative treatment in the treatment of Gradenigo's syndrome resulting from chronic ear disease, we here demonstrate successful conservative treatment of Gradenigo's syndrome.

## 1. Introduction

Chronic suppurative otitis media (CSOM) is characterized by recurrent or persistent ear discharge (otorrhoea) over 12 weeks through a perforation of the tympanic membrane [1]. The complications of CSOM have been greatly reduced because of the development of antibiotics. Complications of CSOM are classified into intracranial and extracranial complications. Extracranial complications can be further divided into intratemporal and extratemporal complications. Different prevalence rates for extracranial complications of CSOM have been reported to date [2–4]. There are still a number of patients, who develop acute mastoiditis, subperiosteal abscess, facial palsy, and intracranial complication due to recent increase of antibiotic-resistant bacteria [5], and other very rare complications like labyrinthitis and petrositis are also encountered. In 1907, Guisseppe Gradenigo described a symptom complex of suppurative otitis media, pain in the distribution of the trigeminal nerve, and abducens (6th) nerve palsy [6]. Gradenigo's syndrome (GS) is defined as a clinical triad of otitis media, severe pain originating from the trigeminal nerve, and ipsilateral sixth cranial nerve palsy. The syndrome is an exceedingly rare complication of CSOM in the era of the widespread use of antibiotics and easily accessible health-care services. Petrositis diagnosis requires imaging studies, namely, computed tomography (CT), magnetic resonance imaging (MRI), or nuclear imaging techniques, to identify the petrous apex as the site of the inflammatory process [3]. The treatment of Gradenigo's syndrome is not consensual and should be managed on an individual basis [7].

Here, we reported a case of acute labyrinthitis and Gradenigo's syndrome following CSOM, treated successfully with conservative therapy with antibiotics, and there was also accidental finding of an internal auditory canal lesion most likely acoustic neuroma.

## 2. Case Report

**Figure 1:**
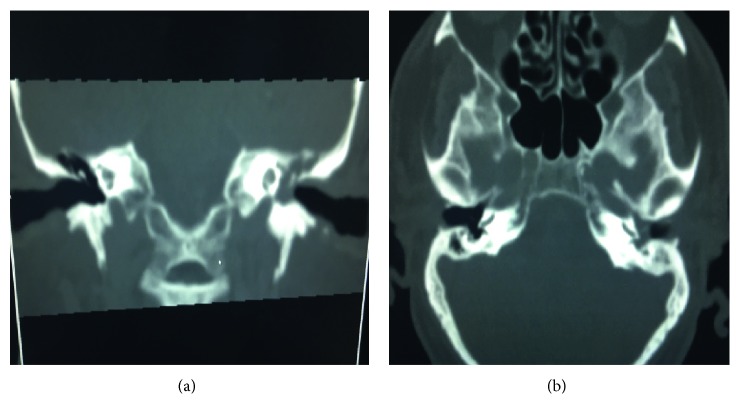
CT scan of temporal bone showing features of tympanomastoiditis and soft tissue shadow involving the middle ear and attic areas. (a) Coronal. (b) Axial.

A 61-year-old man referred from the emergency department to the ear, nose, and throat (ENT) clinic in Al Wakra Hospital because of vertigo and left ear discharge. The vertigo is rotatory in nature and is associated with hearing impairment and tinnitus as well as nausea and vomiting. Ear discharge was purulent, odorless, and intermittent for the last few years, but it became profuse and continuous for the last few days. The described symptoms were associated with severe left-sided headache and diplopia, and there were associated medical comorbidities (diabetic and hypertensive patient). On examination, the patient was conscious, oriented, and not feverish. Left ear examination showed pulsating purulent discharge with granulation tissue filling the middle ear cavity, the tympanic membrane was perforated, and the fistula test was negative. There was left beating nystagmus with left sixth cranial nerve palsy. Other ENT and neurological examinations were not remarkable. Pure tone audiometry showed left-sided severe mixed deafness, and left ear swab for microbiological study for culture and sensitivity was negative. Urgent CT scan was done to rule out intracranial complications, and it showed features of tympanomastoiditis and soft tissue shadow involving the middle ear and attic areas (Figures 1(a) and 1(b)).

**Figure 2:**
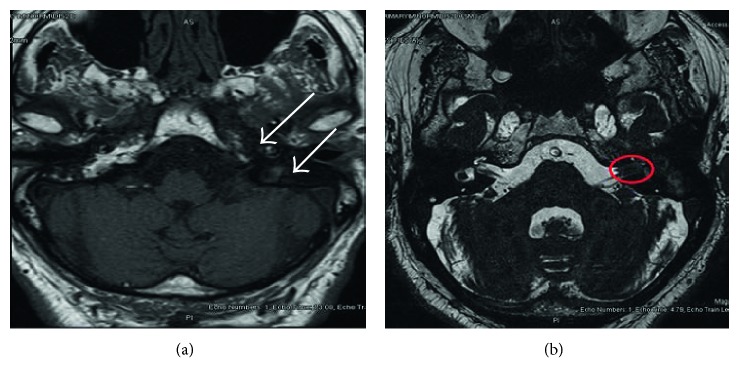
(a) MRI with contrast showing asymmetrical signal changes in the bilateral petrous bone with reduced enhancement on the left with high suspicion of petrositis. (b) Contrast showing an enhancing lesion in the internal auditory meatus involving the 7th-8th nerve complex most likely acoustic neuroma (red circle).

MRI with contrast showed asymmetrical signal changes in the bilateral petrous bone with reduced enhancement on the left with high suspicion of petrositis, in the context of chronic tympanomastoiditis (Figure 2(a)).

In addition to the mentioned pathology, there was a 10 × 4 mm enhancing lesion in the internal auditory meatus involving the 7th-8th nerve complex most likely acoustic neuroma, and there was no extension to the cerebellopontine angle (Figure 2(b)). Conservative treatment started with local and parenteral antimicrobial agents with labyrinthine sedative drugs. After ten-day treatment with good monitoring of blood sugar, the patient had satisfactory response and improvement regarding symptoms of ear discharge, vertigo, and diplopia, but there is no remarkable response regarding hearing loss and tinnitus. The patient continued on conservative treatment for the coming two months, he developed symptomatic response regarding vertigo, diplopia, and ear discharge, and further appointment given for exploration of mastoid and middle ear. Exploration of the mastoid and middle ear showed tympanomastoiditis with a lot of granulation tissue in the middle ear, mastoid, and attic; canal wall down procedure was done with successful result after postoperative follow-up.

## 3. Discussion

The prevalence of CSOM is estimated at 2.5–29.5% [8]. Complications of CSOM can cause grave morbidity and even mortality even though there are intratemporal and/or intracranial complications. Infectious ear diseases have become rarer with the advent of broad-spectrum antibiotics. Gradenigo's syndrome (GS) is an uncommon but life-threatening complication of otitis media. The typical presentation of GS comprises a sixth cranial nerve palsy, otorrhoea, headache, and pain along the distribution of the trigeminal nerve. In our patient, there were more than one complication of CSOM, and they were extracranial intratemporal, that is, petrositis and labyrinthitis. MRI and CT are required to identify the deep seated petrous apex as the site of the inflammatory process [9]. While CT scans may demonstrate opacification of the air cells of the petrous apex with cortical bone erosion, MRI is very useful for assessing inflammatory soft tissue lesions around the petrous apex [10]. Both CT and MRI are essential to establish opacification of air cells in the petrous apex under suspicion, as opposed to asymmetric pneumatization. In our patient, both the CT scan and MRI were done, but MRI was more helpful in the diagnosis of petrositis; otherwise, the labyrinthitis in both facilities would not show any remarkable findings. This is probably because there was circumscribe labyrinthitis, and there was no involvement of bony and membranous labyrinth. Cases of GS as a complication of acute otitis media have usually been successfully treated with broad-spectrum antibiotics, even in cases of petrous abscess formation [11]. In the treatment of chronic ear disease, most authors support surgical intervention as primary management to ensure adequate petrous and mastoid drainage [12, 13]. However, a case of successful conservative treatment of GS associated with chronic otitis media was reported with the use of antibiotic therapy [14, 15]. Our case involved GS and labyrinthitis as complications of chronic ear disease, in which symptoms and sign responded primarily, completely, and successfully to antibiotics therapy. However, the patient explored later on for tympanomastoiditis disease. Regarding the hearing loss and tinnitus, there was no remarkable response, and the explanation could be related to pressure due to pathology that cause an enhancing lesion in the internal auditory meatus involving the 7th-8th nerve complex most likely acoustic neuroma.

## 4. Conclusion

Although there is little evidence to support the use of medical therapy in the treatment of Gradenigo's syndrome resulting from chronic ear disease, we here demonstrate successful conservative treatment of Gradenigo's syndrome and labyrinthitis.
